# Insight into the Effect of Selenization Temperature for Highly Efficient Ni-Doped Cu_2_ZnSn(S,Se)_4_ Solar Cells

**DOI:** 10.3390/nano12172942

**Published:** 2022-08-26

**Authors:** Fancong Zeng, Yingrui Sui, Meiling Ma, Na Zhao, Tianyue Wang, Zhanwu Wang, Lili Yang, Fengyou Wang, Huanan Li, Bin Yao

**Affiliations:** 1 Key Laboratory of Functional Materials Physics and Chemistry of the Ministry of Education, Jilin Normal University, Siping 136000, China; 2 State Key Laboratory of Superhard Materials, College of Physics, Jilin University, Changchun 130012, China

**Keywords:** solar cell, selenidation temperature, CNZTSSe, sol gel

## Abstract

Cu_2_Ni_0_·_05_Zn_0_·_95_Sn(S,Se)_4_ (CNZTSSe) films were synthesized on Mo-coated glass substrates by the simple sol–gel means combined with the selenization process, and CNZTSSe-based solar cells were successfully prepared. The effects of selenization temperature on the performance and the photoelectric conversion efficiency (PCE) of the solar cells were systematically studied. The results show that the crystallinity of films increases as the selenization temperature raises based on nickel (Ni) doping. When the selenization temperature reached 540 °C, CNZTSSe films with a large grain size and a smooth surface can be obtained. The Se doping level gradually increased, and Se occupied the S position in the lattice as the selenization temperature was increased so that the optical band gap (Eg) of the CNZTSSe film could be adjusted in the range of 1.14 to 1.06 eV. In addition, the Ni doping can inhibit the deep level defect of Sn_Zn_ and the defect cluster [2Cu_Zn_ + Sn_Zn_]. It reduces the carrier recombination path. Finally, at the optimal selenization temperature of 540 °C, the open circuit voltage (V_oc_) of the prepared device reached 344 mV and the PCE reached 5.16%.

## 1. Introduction

In recent years, Cu_2_ZnSn(S,Se)_4_ (CZTSSe) thin films have become a new photovoltaic material. CZTSSe films have p-type conductivity, an ideal optical absorption coefficient of about 10^4^ cm^−1^, and an Eg of 1.0–1.5 eV [1,2,3,4,5,6]. At present, through people’s continuous efforts, the PCE of Cu_2_ZnSnS_4_ (CZTS) solar cells reached a record 12.6% from 0.66% in 2014 years [7]. However, it is still far from the theoretical efficiency. The theoretical PCE of CZTS-based solar cells is up to 32.2% [8,9]. The efficiency of CZTSSe devices has not been further improved at the moment. The key factors are the low V_oc_ and high open voltage loss.

Recent studies have shown that the rich inherent defects in thin-film solar cells will lead to a short minority carrier life and harmful band gap potential fluctuations, thus increasing the loss of open circuit voltage [10,11,12]. To improve this low V_oc_, it is an effective strategy to add isovalent cations into kesterite CZTSSe to replace Cu, Zn, or Sn and decrease harmful inherent defects [13,14,15,16]. In our previous work, we selected Ni ions with the closest radius to Zn ions for equivalent substitution doping, which effectively suppressed the intrinsic defects in the crystal: Cu_Zn_ anti-site defects and (2Cu_zn_ + Sn_Zn_) defect clusters. The experimental results show that the V_oc_ value of CNZTSSe devices with the optimal Ni doping content is 42 mV higher than that of CZTSSe devices. In the process of preparing CNZTSSe devices, the CNZTSSe absorption layer is usually synthesized by a two-step process. First, a CNZTS precursor film with an appropriate element composition is deposited. Then, the precursor films are heat treated in a selenium-rich environment. Consequently, to further obtain high-quality CNZTSSe devices, it is necessary to optimize the selenization conditions of CNZTSSe films [17,18,19].

At present, the influence of the post-annealing temperature on the performances of the films has been reported in many works. For example, Kamoun et al. deposited CZTS by adjusting the post-annealing temperature [20]. They found that the post-annealing temperature is vital to enhance the properties of thin films. The IBM group deposited an undoped CZTSSe absorber through post-annealing treatment at 375 °C to improve equipment efficiency [21]. The results indicate that the post-annealing treatment can effectively enhance the crystal quality of the films [20,21]. So far, the effects of selenization temperature on the performances of CNZTSSe films and solar cells have not been studied and reported in detail.

In this work, we successfully deposited CNZTS precursor films on the Mo-coated glass substrates and the CNZTS precursor films were then post-annealed in a selenization atmosphere. By adjusting the selenization temperature, we systematically explored the influence of selenization temperature on the grain growth, photoelectric performances of CNZTSSe films, and CNZTSSe device efficiency. Our work shows that the selenization temperature has an obvious effect on the crystallinity of the films. The film surface is smooth, compact, and almost without holes at the optimum selenization temperature (540 °C), which can effectively reduce grain boundaries, avoid the recombination of minority carriers at grain boundaries, and improve the carrier concentration. Finally, we successfully prepared CNZTSSe solar cells with a PCE of 5.16% by optimizing the selenization temperature.

## 2. Experimental

### 2.1. Preparation of CNZTSSe Thin Films

Using dimethyl Acer (DMSO) (Aladdin, Shanghai Aladdin Biochemical Technology Co., Ltd., Shanghai, China, 99.99%) as the solvent, C_4_H_6_CuO_4_·H_2_O (Aladdin, Shanghai Aladdin Biochemical Technology Co., Ltd., Shanghai, China, 99.99%), ZnCl_2_ (Aladdin, Shanghai Aladdin Biochemical Technology Co., Ltd., Shanghai, China, 99.99%), NiNO_3_·6H_2_O (Sinopharm Chemical Reagent Co., Ltd. Shanghai, China 99.99%), SnCl_2_·2H_2_O (Aladdin, Shanghai Aladdin Biochemical Technology Co., Ltd., Shanghai, China, 99.99%), and CH_4_N_2_S (Aladdin, Shanghai Aladdin Biochemical Technology Co., Ltd., Shanghai, China, 99.99%) as solutes, the CNZTS precursor solution was obtained by overall dissolution, stirring, and heating for 1 h. Then, the above solution was deposited on the Mo-coated sodium calcium glass (SLG) substrate with a rotating speed of 3000 rpm and a spin coating time of 30 s and then dried on a hot plate with a temperature of 300 °C in a nitrogen atmosphere. This step was repeated 10 times to obtain a film thickness of 1 um as required by our experiment. Finally, the films were annealed at a high temperature in a selenium-rich environment with 130 mg selenium particles (Aladdin, Shanghai Aladdin Biochemical Technology Co., Ltd., Shanghai, China, 99.99%) as the selenium source. The annealing time was 15 min and the annealing temperatures were set to 500, 520, 540, and 560 °C for different samples. The synthesis of CNZTSSe films was finished via natural cooling to room temperature.

### 2.2. Preparation of CNZTSSe Devices

The structure of CNZTSSe solar cells is an SLG/Mo/CNZTSSe/CdS/i-ZnO/ITO/Ag grid. A CdS buffer layer was synthesized on the CNZTSSe layer using chemical bath deposition (CBD), and then ZnO and ITO layers were continuously deposited onto the CdS buffer layer using magnetron sputtering. A thin film silver electrode (~900 nm) was prepared on the ITO layer via thermal evaporation means, and nine devices with an effective area of 0.19 cm^2^ were prepared via mechanical scribing.

### 2.3. Characterization

The structural characteristics of the films were evaluated via X-ray diffraction (XRD) (Rigaku Corporation, Tokyo, Japan) using Cu Ka (λ = 0.15406 nm). A Renishaw system instrument using an excitation wavelength of 514 nm was run to record the Raman spectrum and test the phase purity. Scanning electron microscopy (SEM) (Hitachi, S-4800, JEOL, Tokyo, Japan) was used to observe the surface morphology of the films. The chemical composition of CNZTSSe films was measured via X-ray photoelectron spectroscopy (XPS) (Thermofisher, Waltham, MA, USA). The constituent element contents of CNZTSSe films were determined by energy-dispersive X-ray spectroscopy (EDS) (Hitachi, S-4800, JEOL, Tokyo, Japan). The electrical and optical performances of CNZTSSe films were investigated by the use of a Hall effect measurement system and an ultraviolet-visible-near-infrared spectrophotometer (UV-3101PC, Tokyo, Japan), respectively. External quantum efficiency (EQE) curves were measured by a Zolix solar cell Scan100 measuring instrument (Zolix, Beijing Zhuoli Hanguang Instrument Co., Ltd., Beijing, China). For the PCE measurement of CNZTSSe-based devices, the current density–voltage (J-V) plots were obtained with a Model 91160 Oriel semiconductor parametric analyzer (Model 94043A, Oriel, Australia) under the simulated AM 1.5 total solar radiation (100 mW/cm^2^).

## 3. Results and Discussion

### 3.1. Structure and Morphology of Thin Films

**Figure 1:**
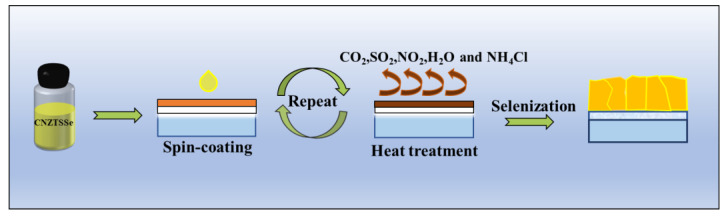
Schematic diagram of CNZTSSe films preparation.

**Figure 2:**
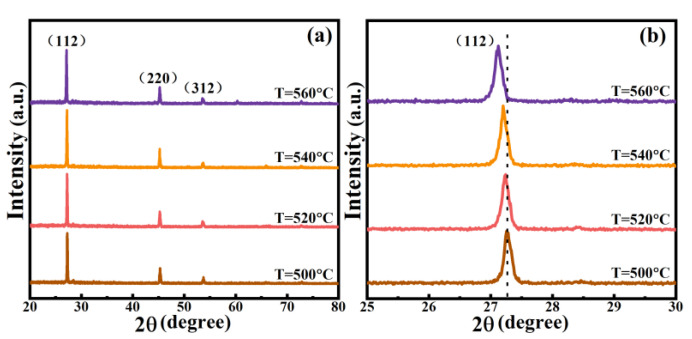
(**a**) XRD patterns of the CNZTSSe films selenized at 500 °C, 520 °C, 540 °C, and 560 °C; (**b**) enlarged images of (112) peaks of all films.

Figure 1 displays the preparation process of the CNZTSSe films. First, a CNZTS film was deposited on SLG using the spin coating method, and then the CNZTSSe films were obtained by selenization annealing. To promote the grain growth of the film during selenization annealing, the selenization temperatures were set to 500, 520, 540, and 560 °C. To reveal the effect of selenization temperature on the crystal structure of the CNZTSSe film, the XRD patterns (Figure 2a) were recorded for the film prepared at various selenization temperatures. There are three main peaks near 2θ = 27.34°, 45.51°, and 53.63° for all films, corresponding to the (112), (220), and (312) crystal planes of CZTSSe, respectively [22,23]. At the same time, no foreign peaks were observed, which means that the selenization temperature has a little effect on the film structure and is not changing the crystal structure of the film. The enlarged (112) peaks are presented in Figure 2b. The change trend can be summarized as follows: with selenization temperature raising, the diffraction angle shifts from 27.25° to 27.12°. This clearly reveals that Se replaces S in the lattice, and this peak position change is owed to the change in unit cell volume caused via the larger Se replacing S in the lattice.

**Figure 3:**
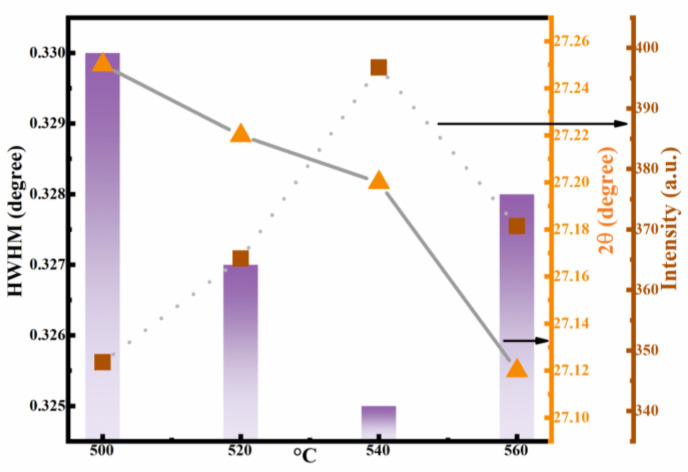
Curves of the 2θ, peak intensity, and FWHM of (112) peaks for the CNZTSSe films as a function of selenization temperature.

To analyze the function of selenization temperature on the grain growth, the curves of 2θ, peak intensity, and half peak width (FWHM) with respect to selenization temperature were drawn by using the test data of XRD. Along with the selenization temperature increasing, the value of 2θ gradually decreased, as shown in Figure 3. Meanwhile, the peak intensity gradually strengthens and the corresponding FWHM decreases. At the optimal selenization temperature (540 °C), the peak intensity is the strongest and the FWHM value is minimal. The results show that the film crystallinity is significantly enhanced. The film crystallinity is very important to obtain ideal thin films. Therefore, it is meaningful to adjust the selenization temperature for obtaining high-quality devices.

**Figure 4:**
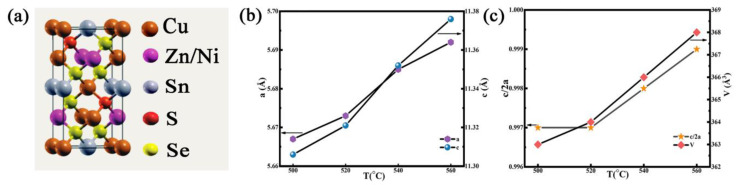
(**a**) Cell structure diagram of CNZTSSe solar cell; (**b**) lattice parameters a and c of all thin film samples; (**c**) unit cell volume (V) and lattice parameters of CNZTSSe thin film (η = c/2a) versus selenization temperature.

The lattice constants a and c, unit cell volume (V), and lattice parameters (η = c/2a) were calculated on the basis of the XRD pattern, as shown in Figure 4b,c. Figure 4a shows a kesterite structure of CNZTSSe. As the selenization temperature is increased, a and c increase, as shown in Figure 4b. Accordingly, V also increases. This is because the larger Se replaces the positions of S in the lattice and the number of Se-substituted S atoms gradually increases, which finally expands the lattice. In Figure 4c, it can be found that all η values are lower than 1. According to the study of Quaternary Chalcogenide Semiconductor structure, η values less than 1 correspond to the crystal structure of the kesterite phase [24]. Therefore, the above results prove that the crystal structure of CNZTSSe films is not changed and it is still a pure kesterite type phase [25].

**Figure 5:**
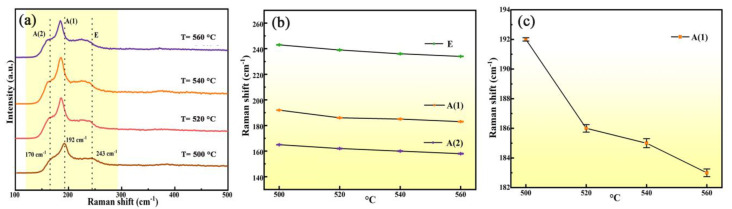
(**a**) Raman spectra of CNZTSSe films prepared at various selenization temperatures; (**b**) variation curve of three vibration mode peaks of CNZTSSe films with selenization temperature; (**c**) variation curve of main Raman vibration mode A (1) with selenization temperature.

Raman measurements are more accurate for analyzing the impurity phases in the films. Because the diffraction peaks of typical secondary phases and CZTSSe phases are very close, it is difficult to distinguish them only by XRD analysis [26]. Figure 5a shows the Raman spectra of all films. It was found that CNZTSSe films show almost the same Raman spectra at different selenization temperatures. All films had three vibration peaks at 170, 192, and 243 cm^−1^. The vibration peaks at 170 and 192 cm^−1^ belong to A-mode Raman vibration peaks of CZTSSe phase, and the vibration peak at 243 cm^−1^ belongs to E vibrational mode Raman peak of CZTSSe phase [27,28]. In addition to the above three vibration peaks, no other vibration peaks were found. This result means that there are no impurity phases in the films. The Raman test results accord well with the above XRD test results. Figure 5b displays the wavenumbers of the three vibration peaks as a function of selenization temperature. The dependence for the main A (1) mode vibration peak is given in Figure 5c. Combining Figure 5b,c, it can be found that the main Raman vibration peak of A (1) has an obvious red shift, and other vibration peaks also have similar changes. The reason for this phenomenon is that the larger Se atom was successfully incorporated into the lattice, replacing the smaller S atom, which correlates well with the XRD results.

**Figure 6:**
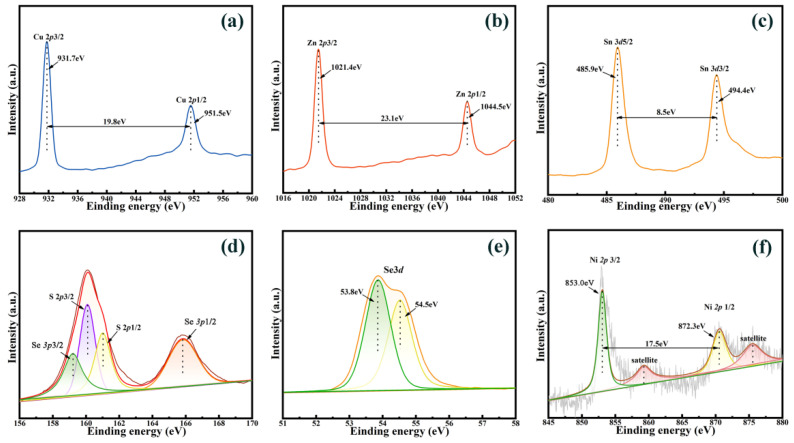
XPS spectra of CNZTSSe films selenized at 540 °C: (**a**) Cu 2p, (**b**) Zn 2p, (**c**) Sn 3d, (**d**) S 2p, (**e**) Se 3d, and (**f**) Ni 2p.

XPS was applied to characterize the composition and the valence states of elements in the CNZTSSe film (540 °C). The representative spectrum of the Cu 2p doublet is presented in Figure 6a. There were two peaks at 931.7 and 951.5 eV, which were identified as Cu 2p3/2 and Cu 2p1/2 components, respectively. The binding energy interval is 19.80 eV, indicating that Cu^+^ exists in CNZTSSe [29]. Figure 6b displays the Zn 2p XPS spectrum, where the Zn 2p3/2 peak was found at 1021.4 eV and the Zn 2p1/2 peak was found at 1044.5 eV. The peak splitting value is 23.1 eV, indicating that Zn exists only in the form of a +2 valence [30]. The XPS spectra of Sn 3d (Figure 6c) indicate two characteristic peaks: Sn 3d5/2 at 485.9 eV and Sn 3d3/2 at 494.4 eV. The splitting value is 8.5 eV, which refers to Sn^4+^ [31]. The XPS spectrum of S 2p is presented in Figure 6d [32,33,34]. The S 2p and Se 3p peak positions are very close. Therefore, the XPS spectrum in Figure 6d shows four characteristic peaks by Gaussian fitting. The characteristic peaks at 159.20, 160.08, 161.02, and 165.84 eV could belong to Se 3p3/2, S 2p3/2, S 2p1/2, and Se 3p1/2, respectively. The peaks at 160.08 eV and 161.02 eV are in accordance with the 160–164 eV range of S^2−^ [35]. As displayed in the Se 3d XPS spectrum (Figure 6e), the peaks at 53.8 and 54.5 eV are attributed to the characteristic peaks of Se 3d3/2 and Se 3d1/2 for the Se^2−^ oxidation state [36,37,38]. Figure 6f shows that the peaks at 853.0 and 872.3 eV are correspondent to Ni 2p3/2 and Ni 2p1/2 components. The binding energy difference between the Ni 2p components is 17.5 eV, and it is in good relation with the splitting value of Ni^2+^ [39,40]. The above experimental results show that the constituent elements of the film exist in the valence states of Cu^+^, Ni^2+^, Zn^2+^, Sn^4+^, S^2−^, and Se^2−^.

The content of the elements in the film was obtained by EDS test, and its content was analyzed according to the test results. Table 1 summarizes the EDS test data of CNZTSSe films selenized at 500, 520, 540, and 560 °C. It can be found that, except for Se and S elements, the contents of other elements and the element content ratio related to Ni have almost no significant changes. It should be noted that the fluctuation in Ni content in the film is very small with the selenization temperature rising, which means that there is no loss of Ni content in the selenization process. This helps to inhibit the formation of Zn-related defects, such as Cu_Zn_ and Sn_Zn_ anti-site defects. In addition, EDS test results indicate that Se content increases by 4% and S content decreases by 2.4% with the selenization temperature rising. This reveals that CNZTSSe films were synthesized under the condition of Se enrichment. At the same time, it also shows that the substitution of the Se atom for the S atom is enhanced. This further supports the conclusion obtained from the XRD results that the Se atom successfully replaces the position of S in the lattice.

**Figure 7:**
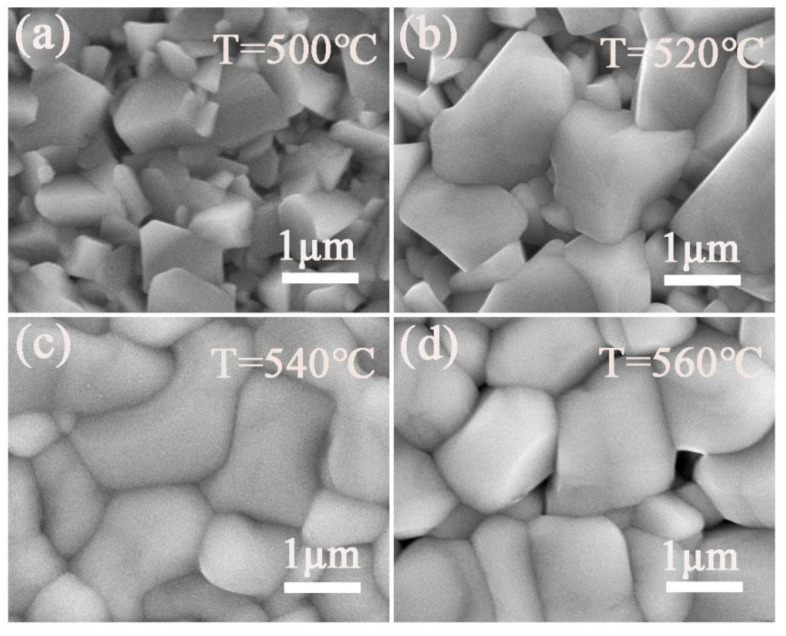
SEM surface images of the CNZTSSe thin films selenized at 500 °C (**a**), 520 °C (**b**), 540 °C (**c**), and 560 °C (**d**).

Figure 7a–d show the SEM images of the top surfaces of the CNZTSSe films selenized at 500, 520, 540, and 560 °C. It can be seen in Figure 7a that when the selenization temperature is 500 °C, the grains are at the initial stage of growth, and they are small. When the selenization temperature rises to 520 °C, the grains grow further, and the grain size becomes larger. However, the small grain layer still exists, and the film’s surface is rough with many voids (Figure 7b). When the selenization temperature is 540 °C, it is obvious that small grains and holes are eliminated. Meanwhile, the grain size reaches micron level, and the morphology of the CNZTSSe film is smooth and dense, as shown in Figure 7c. When the selenization temperature further rose to 560 °C, although the grain size was large, voids began to appear in Figure 7d. The above results show that selenization temperature can promote the grain growth and reduce grain boundaries, which is beneficial to suppress the carrier non-radiative recombination at the grain boundaries [21]. In addition, it is obviously observed that the optimal selenization temperature is 540 °C.

### 3.2. Optical and Electrical Performances of Thin Films

**Figure 8:**
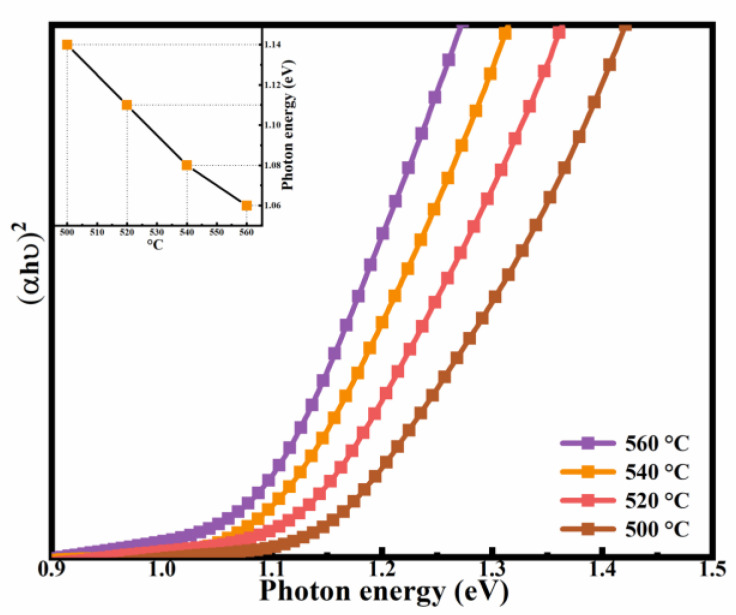
The plot of (αhυ)^2^ against hυ for CNZTSSe films selenized at 500 °C, 520 °C, 540 °C, and 560 °C. The insert shows the Eg values of the CNZTSSe films selenized at 500 °C, 520 °C, 540 °C, and 560 °C.

To determine the influence of selenization temperature on the Eg values in the films, UV-vis-NIR spectra measurements were carried out. The absorption spectra of all samples were obtained, and Figure 8 shows the (αhυ)^2^-hυ curves of the CNZTSSe films. The Eg values can be estimated by processing the optical absorption spectra with the use of the Tauc relation:

(αhυ) = B (hυ − Eg) ^n^ (1)

where α is the absorption coefficient, hυ is the Photon energy, and B is a constant [41,42,43,44]. Theoretical studies show that n corresponds to 1/2, 3/2, 2, and 3 in permitted direct transition, permitted indirect transition, prohibited direct transition, and prohibited indirect transition, respectively [45]. CNZTSSe films are direct bandgap semiconductors, so the value of n is 1/2. Therefore, Equation (2) can be obtained from Equation (1), as follows:

(αhυ) = B (hυ − Eg) ^1/2^ (2)

According to Equation (2), Eg can be gained by inferring the linear part of the curve (αhυ)^2^ as a function of hυ to intercept the energy *x*-axis. When (αhυ)^2^ = 0, Eg = hυ. The Eg values of the CNZTSSe absorption layers selenized at 500, 520, 540, and 560 °C are arranged in the inset of Figure 8. When the selenization temperatures are 500, 520, 540, and 560 °C, the Eg values are equal to 1.14, 1.11, 1.08, and 1.06 eV, respectively. Then, according to the curve given in the inset, it can be found that the value of Eg diminishes with the rising selenization temperature. Everyone knows that the Eg of CZTSe is smaller than that of CZTS [46]. According to the EDS test, as the selenization temperature rises, the content of Se rises and the content of S lessens. Therefore, the Eg of CNZTSSe will be closer to the small Eg of CZTSe as the Se content rises. Moreover, according to the first principal calculation, when Se replaces S, the orbital interaction between the valence band (VBM) top and the conduction band (CBM) bottom is weakened, thus reducing Eg (i.e., CBM = VBM + Eg) [47].

The electrical performances of CNZTSSe films were studied via Hall measurement at room temperature. The test was carried out several times to improve the accuracy of the results. The test results are shown in Table 2. All CNZTSSe films show p-type conductivity. This is mainly ascribed to the typical high concentration of intrinsic Cu_Zn_ anti-site defects in the film, which will make the Fermi level close to VBM, so that the film has p-type conductivity [48]. During the increase in the selenization temperature, the carrier concentration increased from 2.2 × 10^16^ cm^−3^ to 1.15 × 10^17^ cm^−3^ and then reduced to 1.35 × 10^16^ cm^−3^. The corresponding Hall mobility decreased from 1.27 to 1.15 cm^2^V^−1^s^−1^ and then increased to 1.71 cm^2^V^−1^s^−1^. However, at the selenization temperature of 540 °C, the carrier concentration is the highest, which is due to the improvement in film quality, the reduction in the number of grain boundaries, and the reduction in carrier recombination at grain boundaries, thus effectively increasing the carrier concentration. However, when the carrier concentration increases, the carrier scattering will increase, resulting in a decrease in mobility.

### 3.3. Device Performance

**Figure 9:**
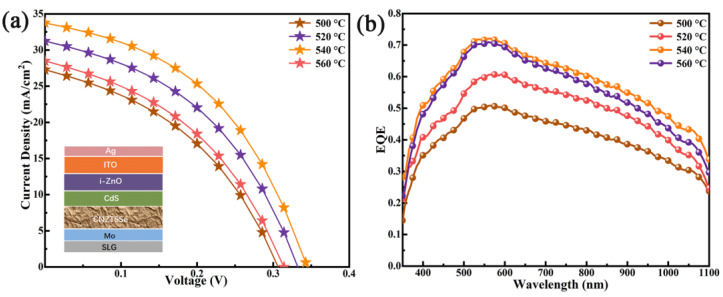
(**a**) Current–voltage characteristics of the CNZTSSe-based solar cells synthesized using the CNZTSSe absorber layer selenized at diverse temperatures of 500 °C, 520 °C, 540 °C, and 560 °C under AM 1.5G illumination; (**b**) EQE spectra of the corresponding CNZTSSe-based solar cells.

**Figure 10:**
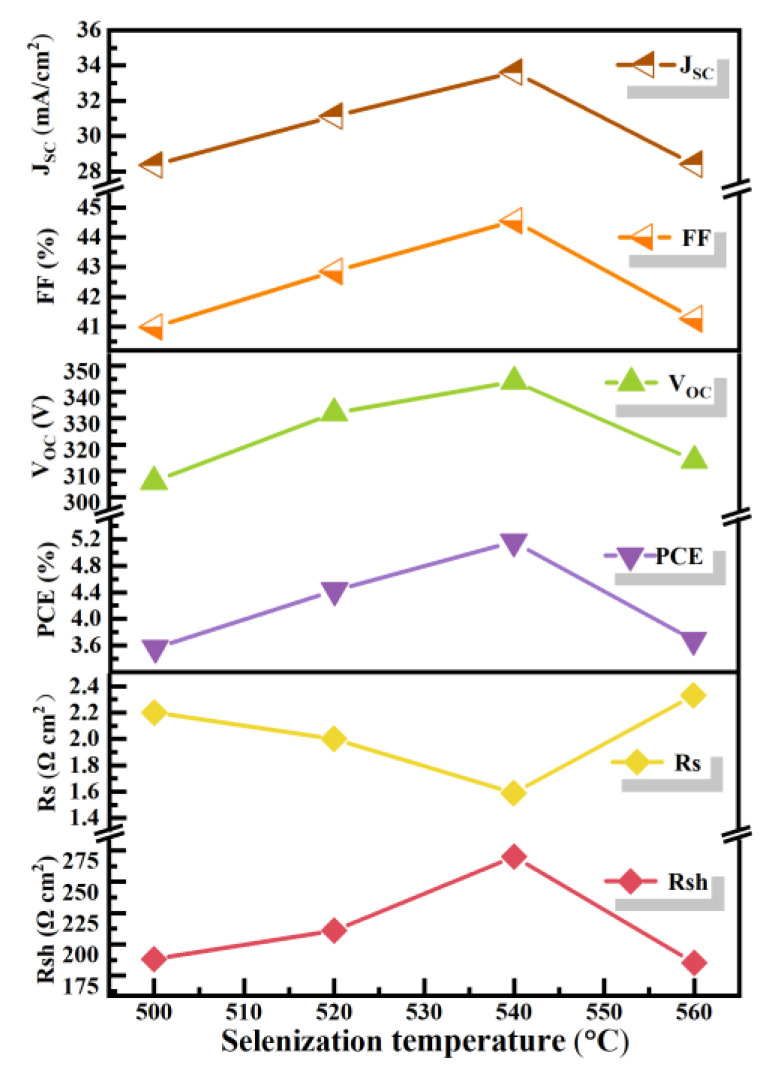
Curves of Jsc, FF, Voc, PCE, Rs, and Rsh as selenization temperature increases.

To reveal the impact mechanism of selenization temperature on the PCE, CNZTSSe devices with traditional structures were prepared by using CNZTSSe as an absorption layer after the selenization at 500, 520, 540, and 560 °C. The main performance parameters of the corresponding devices are listed in Table 3. Figure 9a shows the J-V curves of CNZTSSe solar cells for different selenization temperatures. The layer structure of the prepared CNZTSSe solar cells is given in Figure 9a. Comparing the data presented in Table 3, it can be found that the PCE of the device increases from 3.56 to 5.16% as the selenization temperature rises from 500 to 540 °C, and when the temperature is further increased to 560 °C, the PCE level of the device decreases to 3.68%. Meanwhile, the series resistance (R_s_) of the device gradually decreases until it reaches the minimum value at 540 °C. However, the corresponding shunt resistance (R_sh_) rises with the rising selenization temperature and the selenization temperature corresponding to the maximum value is 540 °C. The decrease in R_s_ can be attributed to the enhancement of film crystallinity and the decrease of crystal boundaries, which reduces the recombination rate of carriers at the grain boundaries. The increase in R_sh_ is due to the smooth and dense CNZTSSe film providing a good substrate for the deposition of the CdS layer so that CdS can be deposited evenly on the surface of the CNZTSSe film and reduce the recombination of carriers at the interface. The R_s_ lessens and R_sh_ increases, leading to the increase in the short circuit current density (J_sc_) and filling factor (FF), which finally leads to the increase in V_oc_. The variation curves of the above main parameters are shown in Figure 10. To sum up, we find that the champion PCE is 5.16% for the device with V_oc_ of 344 mV, J_sc_ of 33.63 mA/cm^2^, and FF of 44.56% when the selenization temperature is 540 °C.

Figure 9b displays the EQE levels of CNZTSSe devices for different selenization temperatures. The results show that the EQE of the device increases significantly in the whole wavelength range from 350 to 1100 nm when the selenization temperature rises from 500 to 540 °C. The improvement of EQE is mainly due to the optimization of the crystal quality of the film, allowing more photons to be absorbed [49]. In addition, the improvement in the EQE shows that the carrier recombination is reduced and the charge collection in the space charge region is enhanced [50]. Yet, when the selenization temperature further rose to 560 °C, the EQE spectral response decreased. This may be caused by the following two aspects. For one thing, the decomposition of the CNZTSSe phase may occur at higher selenization temperatures. For another, due to the film roughness increase at higher selenization temperatures, the formation of a poor PN junction may occur [51]. The above results show that the EQE response of the structure is the most significant at the optimal selenization temperature (540 °C).

## 4. Conclusions

In summary, the single-phase kesterite CNZTSSe thin films were obtained by the simple sol–gel method and selenization process in the temperature range of 500–560 °C. The results show that PCE can be increased from 3.56 to 5.16% by regulating the selenization temperature of CNZTSSe films. The increase in PCE is mainly owed to the increase in V_oc_, which derives from the decrease in Eg and the increase in FF and J_sc_. The PCE changes with the selenization temperature and the PCE can be up to a maximum value of 5.16% for solar cells synthesized using the CNZTSSe absorption layer film selenized at 540 °C. It is proven that the enhancement of PCE with selenization temperature is mainly attributed to the decrease in R_s_ and the increase in R_sh_. The improvement in R_s_ and R_sh_ depends on the enhancement of the crystal quality and grain size of the CNZTSSe film. In addition, Ni doping in CZTSSe films can alleviate the negative effects of defects from Sn_Zn_ and [2Cu_Zn_ + Sn_Zn_] on devices at 540 °C, which reduces the carrier recombination rate and increases the carrier concentration.

## Figures and Tables

**Table 1:** EDS results of the CNZTSSe films selenized at various temperatures.

Sample	Cu (% at)	Ni (% at)	Zn (% at)	Sn (% at)	S (% at)	Se (% at)	Cu/(Zn + Ni + Sn)	(Ni + Zn)/Sn
500	23.81	0.34	16.28	10.98	2.80	45.79	0.862	1.513
520	23.75	0.36	16.26	10.97	2.47	46.22	0.860	1.515
540	23.79	0.35	16.30	10.99	2.06	46.51	0.860	1.515
560	23.74	0.36	16.25	10.95	1.78	46.91	0.861	1.516

**Table 2:** Electrical properties of the CNZTSSe films selenized as the selenization temperature of 500 °C, 520 °C, 540 °C, and 560 °C.

Samples	Resistivity (Ω·cm)	Carrier Concentration (cm^−3^)	Mobility (cm^2^V^−1^s^−1^)	Type
T = 500 °C	1.65 × 10^2^	2.25 × 10^16^	1.27	p
T = 520 °C	1.63 × 10^2^	2.63 × 10^16^	1.43	p
T = 540 °C	1.52 × 10^2^	1.15 × 10^17^	1.15	p
T = 560 °C	2.70 × 10^2^	1.35 × 10^16^	1.71	p

**Table 3:** Performance parameters of devices selenized at various selenization temperatures.

Device	Active Area	V_OC_ (mV)	J_SC_ (mA/cm^2^)	FF (%)	PCE (%)	R_s_ (Ω cm^2^)	R_sh_ (Ω cm^2^)
CNZTSSe (T = 500 °C)	0.19 cm^2^	306	28.37	40.98	3.56	2.20	188
CNZTSSe (T = 520 °C)	0.19 cm^2^	332	31.14	42.85	4.43	2.00	211
CNZTSSe (T = 540 °C)	0.19 cm^2^	344	33.63	44.56	5.16	1.59	270
CNZTSSe (T = 560 °C)	0.19 cm^2^	314	28.41	41.26	3.68	2.33	185
